# Mechanism of metamifop resistance in *Digitaria ciliaris* var. *chrysoblephara* from Jiangsu, China

**DOI:** 10.3389/fpls.2023.1133798

**Published:** 2023-02-21

**Authors:** Jingjing Cao, Yuan Tao, Zichang Zhang, Tao Gu, Gui Li, Yuanlai Lou, Hongchun Wang

**Affiliations:** Institute of Plant Protection, Jiangsu Academy of Agricultural Sciences, Nanjing, Jiangsu, China

**Keywords:** *Digitaria ciliaris* var. *chrysoblephara*, metamifop, weed resistance, target-site resistance, cross- and multi-resistance

## Abstract

*Digitaria ciliaris* var. *chrysoblephara* is one of the most competitive and problematic grass weeds in China. Metamifop is an aryloxyphenoxypropionate (APP) herbicide that inhibits the activity of acetyl-CoA carboxylase (ACCase) of sensitive weeds. Following the introduction of metamifop to China in 2010, it has been continuously used in rice paddy fields, thereby substantially increasing selective pressure for resistant *D*. *ciliaris* var. *chrysoblephara* variants. Here, populations of *D*. *ciliaris* var. *chrysoblephara* (JYX-8, JTX-98, and JTX-99) were observed to be highly resistant to metamifop, with resistance index (RI) values of 30.64, 14.38, and 23.19, respectively. Comparison of resistant and sensitive population ACCase gene sequences revealed that a single nucleotide substitution from TGG to TGC resulted in an amino acid substitution from tryptophan to cysteine at position 2,027 in the JYX-8 population. No corresponding substitution was observed for JTX-98 and JTX-99 populations. The ACCase cDNA of *D*. *ciliaris* var. *chrysoblephara* was successfully obtained by PCR and RACE methods, representing the first amplification of full length ACCase cDNA from *Digitaria* spp. Investigation of the relative expressions of ACCase gene revealed the lack of significant differences between sensitive and resistant populations before and after herbicide treatments. ACCase activities in resistant populations were less inhibited than in sensitive populations and recovered to the same or even higher levels compared to untreated plants. Whole-plant bioassays were also conducted to assess resistance to other ACCase inhibitors, acetolactate synthase (ALS) inhibitors, auxin mimic herbicide, and protoporphyrinogen oxidase (PPO) inhibitor. Cross-resistance and some multi-resistance were observed in the metamifop-resistant populations. This study is the first to focus on the herbicide resistance of *D*. *ciliaris* var. *chrysoblephara*. These results provide evidence for a target-site resistance mechanism in metamifop-resistant *D*. *ciliaris* var. *chrysoblephara*, while providing a better understanding of cross- and multi-resistance characteristics of resistant populations that will help in the management of herbicide-resistant *D*. *ciliaris* var. *chrysoblephara*.

## 1 Introduction

*Digitaria* spp. are annual gramineae weeds that infest turfgrass, roadsides, wastelands, and crop systems like *Zea mays*, *Glycine max*, and *Saccharum officinarum*, among others (Basak et al., 2019). They are globally widely distributed in tropical, subtropical, and temperate regions, including in the eastern, northern, northwestern, and northeastern areas of China. Labor shortages and agricultural mechanization have led to the popularization of dry direct-seeding rice cultivation due to its high potentials for resource conservation and economic returns (Matloob et al., 2015; Rao et al., 2017). Dry direct-seeding rice cultivation accounts for over 35% of rice cultivation in China and has become one of the primary methods of rice cultivation in the Jiangsu Province. Dry-wet changes of soils in dry direct-seeding rice fields benefit weeds growth. Various weeds are present in fields that have larger occurrence and longer symbiotic times, thereby seriously threatening the yields and quality of rice. *Digitaria* spp. weeds have consequently become the dominant weeds in dry direct-seeding rice fields of the Jiangsu Province due to their adaptation to changes in local cropping systems and ecological environments (Wang et al., 2019).

Acetyl-CoA carboxylase (ACCase) inhibitors are one of the most widely used and important classes of herbicides used to control grass in rice fields. ACCase inhibitors can be divided into three types based on differences in their active components, including aryloxyphenoxypropionate (APP), cyclohexenone (CHD), and phenylpyrazole (PPZ) types (Fang et al., 2020). Plant ACCase catalyzes the carboxylation of acetyl-CoA to malonyl-CoA, thereby providing substrates for the synthesis of fatty acids and numerous secondary metabolites (Adina-Zada et al., 2012). Two forms of ACCase are typically present in plants, including heteromeric and homomeric forms (Takano et al., 2020). Poaceae plants possess homomeric ACCase, while dicotyledonous plants have homomeric form in the cytoplasm and heteromeric form in the plastids. Consequently, ACCase-inhibiting herbicides exhibit specific activities against gramineae plants that can selectively inhibit homomeric plastidic ACCase only found in monocots, but do not inhibit heteromeric plastic nor homomeric cytosolic forms, allowing dicots to become tolerant to them (Kukorelli et al., 2013; Takano et al., 2020).

Previous studies have shown that the application of ACCase inhibitors leads to the gradual development of resistance after continuous use over 6 to 10 years (Huang et al., 2003), suggesting a relatively high risk of herbicide resistance. Mechanisms of weed resistance can be divided into target-site resistance (TSR) and non-target-site resistance (NTSR). TSR to ACCase inhibitors is primarily associated with changes in amino acids caused by mutations of ACCase gene, leading to changes in proteins that bind to herbicides, and ultimately leading to the resistance of weeds to herbicides. Sixteen amino acid substitutions at seven sites have been identified in the carboxyl-transferase (CT) domain of plastidic ACCase, including: Ile-1781-Leu, Ile-1781-Val, Ile-1781-Thr, Trp-1999-Cys, Trp-1999-Leu, Trp-1999-Ser, Trp-2027-Cys, Trp-2027-Ser, Ile-2041-Asn, Ile-2041-Val, Ile-2041-Thr, Asp-2078-Gly, Asp-2078-Glu, Cys-2088-Arg, and Gly-2096-Ala, and Gly-2096-Ser (Collavo et al., 2011; Kaundun et al., 2013a; Kaundun et al., 2013b; Papapanagiotou et al., 2015; Guo et al., 2017; Li et al., 2017; Wang et al., 2021). The overexpression of ACCase gene was also identified as a TSR mechanism that effectively increases the amount of target protein to mitigate the toxic effects of herbicide on weeds, as has been primarily shown for glyphosate resistance (Kupper et al., 2017; Zhang et al., 2018). However, few studies have evaluated ACCase gene expression involved in herbicide resistance. Notably, Laforest et al. observed that the overexpression of ACCase gene led to the resistance of *Digitaria sanguinalis* to ACCase inhibitors (Laforest et al., 2017). NTSR mechanisms are more complicated and include reduced penetration and translocation, sequestration, and enhanced herbicide metabolism (Delye et al., 2011a). These mechanisms can confer complex cross-resistance to herbicides *via* different modes of action (Yu and Powles, 2014). Further, various enzymes are involved in conferring non-target-site resistance to herbicides, including cytochrome P450 monooxygenases and gluthathione-S-transferases (Brazier et al., 2002).

Metamifop is a type of APP herbicide that has been used continuously following its introduction to China. The herbicide features advantages of low toxicity, high efficiency, environmental safety, and excellent miscibility, among other characteristics. It is a common agent used to control most annual gramineae weeds and exhibits good control of *Digitaria* spp. weeds. Continuous and extensive use of metamifop has led to increased selection pressures on weeds, eventually leading to a serious risk of herbicide resistance. Analogously, *Echinochloa* spp. and *Leptochloa chinensis* have developed resistance to metamifop in China and elsewhere (Won et al., 2014). Mutations of ACCase gene corresponding to Ile-1781-Leu, Trp-1999-Ser, and Trp-2027-Cys are the most common TSR mechanisms identified in metamifop-resistant *Echinochloa* spp. in China. Furthermore, NTSR mechanisms of *Echinochloa crus*-*galli* to metamifop have also been identified (Xia et al., 2016). Moreover, it has been reported that *Leptochloa chinensis* developed resistance to metamifop due to the ACCase gene mutation Trp-2027-Ser (Yuan et al., 2021). Few studies have evaluated metamifop resistance in *D*. *ciliaris*. Yu et al. observed that *D*. *ciliaris* can be resistant to sethoxydim and cross-resistant to fenoxaprop and fluazifop (Yu et al., 2017). Further, Basak et al. observed that the Ile-1781-Leu mutation of the ACCase gene led to pinoxaden resistance (Basak et al., 2019). However, herbicide-resistant *D*. *ciliaris* var. *chrysoblephara* populations have not yet been observed in China.

In this study, three *D*. *ciliaris* var. *chrysoblephara* populations that are resistant to metamifop were collected from dry direct seeding rice fields in Jiangsu Province, China. The aims of the present study were consequently to: (1) determine the level of resistance to metamifop in putatively resistant *D*. *ciliaris* var. *chrysoblephara* populations; (2) identify the mechanisms responsible for metamifop resistance in *D*. *ciliaris* var. *chrysoblephara* based on ACCase gene sequencing, gene expression, and enzyme activity; and (3) characterize cross- and multi-resistance of the resistant populations to various herbicides with different modes of action.

## 2 Materials and methods

### 2.1 Plant materials

Seeds of *D*. *ciliaris* var. *chrysoblephara* suspected of being resistant to metamifop were collected from dry direct-sown rice fields in October 2020 (**Table 1**). Seeds of plants from each field were pooled to represent a population. JYX-8, JTX-98, and JTX-99 were presumed to be resistant populations and JSS-19 was presumed to be a sensitive population based on single dose assay pre-experiments. *D*. *ciliaris* var. *chrysoblephara* seeds were germinated on filter paper in Petri dishes in June 2021 over a 12 h photoperiod with 2,500 xL lighting and incubation at 30/25°C for several days. Fifteen seedlings were then transplanted to a plastic pot (9.5 cm diameter and 16 cm height) containing loam soil and grown in an incubator under the above-mentioned conditions.

**Table 1:** *D. ciliaris* var. *chrysoblephara* seed collection information.

Populations	Collection Site	Collection Time
JSS-19	Sucheng, Suqian, Jiangsu	2020.9
JYX-8	Xiangshui, Yancheng, Jiangsu	2020.9
JTX-98	Xinghua, Taizhou, Jiangsu	2020.9
JTX-99	Xinghua, Taizhou, Jiangsu	2020.9

### 2.2 Dose response to metamifop

Dose response experiments were performed when the seedlings reached the four-leaf stage. Presumed resistant and sensitive populations were sprayed using a 3WPSH-500D type bioassay spray tower (Nanjing Institute of Agricultural Mechanization, Ministry of Agriculture and Rural Affairs) using a disc diameter of 50 cm, a nozzle diameter of 0.3 mm, a spray pressure of 0.3 MPa, a droplet diameter of 100 µm, and a sprinkler flow of 90 mL·min^−1^. Metamifop doses of 0-, 0.03125-, 0.0625-, 0.125-, 0.25-, 0.5-, 1-, and 2-fold the recommended dose (120 g a.i ha^−1^) were applied to the sensitive population, while 0-, 0.25-, 0.5-, 1-, 2-, 4-, 8-, and 16-fold the recommended dose were applied to the resistant populations. Each pot with 15 seedlings was set up as a replicate. Each treatment comprised four replicates and the experiment was conducted twice.

The above-ground fresh weights of plants were measured 21 days after treatment. Data were pooled since no significant differences between the two repeated experiments were identified based on *t*-test (*p*< 0.05) by ANOVA (SPSS v.16.0). The SigmaPlot 12.0 software was used to calculate the GR_50_ values (herbicide dose causing 50% growth reduction) for different populations of *D*. *ciliaris* var. *chrysoblephara*. A log-logistic model was used to analyze the test data, with the fitting equation as follows:


$$y=c+\left(d-c\right)/\left[1+{\left(x/GR_{50}\right)}^{b}\right]$$

In the equation, *y* is the fresh weight percentage of the control, *c* is the lower limit, *d* is the upper limit, *b* is the slope, and *x* is the herbicide dose (Seefeldt et al., 1995). The resistance index (RI) was obtained based on the GR_50_ values of the resistant population/GR_50_ of the sensitive population.

### 2.3 ACCase CT domain and cDNA sequencing

Young leaves from individual plants at the four-leaf stage were subjected to total DNA extraction using a plant genomic DNA extraction kit (Tiangen, China), according to the manufacturer’s instructions. The conserved CT domain of ACCase gene was then amplified using two pairs of the previously designed universal primers ACcp1/ACcp1R and ACcp4/ACcp2R (Delye et al., 2011b). PCR reaction volumes were 50 µL and included 2 µL of DNA, 2 µL of forward and reverse primers (10 µM), 25 µL of 2×PCR long Taq Mix (Vazyme, China), and ddH_2_O up to 50 µL. Reactions included a pre-denaturation step of 5 min at 95°C, followed by 30 cycles at 94°C for 30 s, 58°C for 30 s, and 72°C for 30 s. The PCR products were subject to electrophoresis in a 0.75% agarose gel in 1×TAE buffer, and PCR fragments were cloned into the PMD-18T vector (Takara, China) for sequencing. Amplicons from individual plants in each population were sequenced and at least 10 clones from each plant were subjected to sequencing to construct ACCase consensus sequences. Each segment was sequenced in the forward and reverse directions at Invitgen Biotechnology, Ltd. (Shanghai, China) to reduce sequencing errors. The BioEdit sequence alignment editor software was used to align and compare sequence data.

Total RNA was extracted using the RNApre Pure Plant Kit, followed by first strand cDNA generation with the FastQuant RT Kit (Tiangen, China). ACCase cDNA gene sequences of gramineae plants with high homology to *D*. *ciliaris* var. *chrysoblephara* were downloaded from NCBI, including those from *Beckmannia syzigachne* (GenBank accession number: KF501575), *Alopecurus myosuroides* (GenBank accession number: AJ310767) and *Lolium rigidum* (GenBank accession number: AY995232). The sequences were compared with the DNAMAN software. Eight pairs of primers were designed based on homologous sequence comparison of conserved regions (**Table 2**). In addition, 5’ cDNA terminal rapid amplification (RACE) gene specific primers (**Table 2**) were designed, and the ACCase cDNA ends were amplified using the HiScript-TS 5’/3’ RACE Kit (Vazyme, China). PCR fragments amplified from resistant populations were cloned into the PMD-18T vector (Takara, China) and amplicons from ten plants of each population were sequenced. For each biological replicate, at least ten clones were sequenced and used to construct ACCase consensus sequences. The BioEdit sequence alignment editor and DNAMAN software were then used to analyze and compare the sequence data.

**Table 2:** PCR primers used to amplify ACCase cDNA of *D*. *ciliaris* var. *chrysoblephara*.

Primer	Sequence (5’to 3’)	Annealing temperature (°C)	Gene location
5GSP	TCTCCTCAGGTATCGAGTCC	For nested PCR	For nested PCR
5GNP	AGGCCATCCAAGAATCAAAG		
A.C-F2	TTCAGCTCTCATTGCTCAAG	59.3	777–1,971
A.C-R2	TAGAGCTCCTCCAACCACTG		
A.C-F3	ATTCAAATTCGTGGAGAAAT	55.8	1,801–3,153
A.C-R3	AGCTTTGTTCCTCACACCCT		
A.C-F4	TTTTCAGTGATGGCATTCAG	57.6	3,038–4,052
A.C-R4	CGTGATGGAGTATACTTCAT		
A.C-F5	AGATCAGATTCTCCGGCATG	56.4	3,951–5,031
A.C-R5	TGGGCCAAATGATCCAGCTC		
A.C-F6	TTACTAGTCACACCTGTACAG	57.6	4,544–5,683
A.C-R6	TAGGAAGAGGTCCACCAATG		
A.C-F7	GACTGTTTCAGATGACCTTG	55.9	5,598–6,631
A.C-R7	AAGGATATCTGAAGATGTTC		
A.C-F8	TGTTATGCTGAGAGGACTG	56.0	6,259–6,966
A.C-R8	CAGCCGCCTTGTATCCATCT		

### 2.4 ACCase gene expression assay

After growing to the four-leaf stage, plant seedlings from four *D*. *ciliaris* var. *chrysoblephara* populations were sprayed with the recommended dose of metamifop (120 g a.i.ha^−1^). Seedlings from each population were collected at 0, 12, and 24 h after treatment. Tissues were subjected to RNA extraction and cDNA synthesis, as described above. Primers were designed with the Primer premier 5.0 software package (AC-F: CTGTTGTGGGCAAGGAGGATG; AC-R: TACCAAGCCGAGCAAGATAAG) to amplify 153 bp fragments of target ACCase gene. Actin was used as the internal reference gene and amplified using primers designed using the large crabgrass actin gene (GenBank: KY967696) (Actin-F1: CGGAGAATAGCATGAGGAAGTG; Actin-R1: AGTGGTCGAACAACTGGTATTG). Real-time PCR was conducted with an ABI QuantStudio™ 7 Flex Real-Time PCR System (Thermofisher, USA) using a 2×TSINGKE Master qPCR Mix (SYBR Green I) kit (Tsingke, China). Thermal cycling conditions were used according to the manufacturer’s instructions. Three biological replicates and four technical replicates were used for each sample. The relative expression of ACCase gene were calculated using the 2^−ΔΔCT^ method, while expression from the untreated sensitive population was used as the negative control.

### 2.5 ACCase activity assay

After growing to the four-leaf stage, seedlings of four *D*. *ciliaris* var. *chrysoblephara* populations were sprayed with the recommended dose of metamifop (120 g a.i.ha^−1^). Seedlings were collected from each population at 0, 2, 12, 24, 48, and 72 h after treatment, respectively. ACCase catalyzes the transformation of Acetyl-CoA, NaHCO_3_, and ATP to generate Malonyl-CoA, ADP, and inorganic phosphorus. The interaction of molybdenum blue and phosphate can generate products with characteristic absorption peaks at 660 nm. Thus, ACCase activity was determined based on inorganic phosphorus levels using the ammonium molybdate method. Specifically, ACCase activity was quantified using an ACCase activity assay kit (Biobox, China) and a microplate spectrophotometer (Agilent BioTek Epoch2, USA). Crude enzyme was prepared by adding 1 mL of extracting solution to 0.1 g of leaf tissue. The enzymatic reactions and phosphate quantification were then conducted based on the manufacturer’s instructions. Absorbance values at 660 nm were determined for experimental reactions, in addition to those for negative controls, blank controls, and to establish a standard curve. Three biological replicates and three technical replicates were used for each sample.

The unit of ACCase activity was calculated based on sample mass, as defined by the amount of 1 μmol of inorganic phosphorus generated for 1 g tissue over 1 h. Specifically, ACCase activity was calculated with the following equation:


$$\mathrm{ACCase}\;\mathrm{activity}\left(U/g\;\mathrm{mass}\right)=x{*}_{V}\mathrm{total}/\left[\left(V_{\mathrm{sample}}*W\right)/V_{\mathrm{total}\;\mathrm{sample}}\right)]/T=20x/W$$

In the formula, *V_total_* is the total volume of the enzymatic reaction (0.1 mL), *V_sample_* is the volume of added sample (0.01 mL), *V_total sample_* is the volume of extracting solution (1 mL), *T* is the time of enzymatic reaction (0.5 h), and *W* is the fresh weight of sample (0.1 g).

### 2.6 Cross- and multi-resistance to other herbicides

Dose-response experiments were performed to determine whether the *D*. *ciliaris* var. *chrysoblephara* populations exhibited cross- or multi-resistance to other herbicides. Experiments were described as in section 2.2, and the other evaluated herbicides are listed in **Table 3**.

**Table 3:** Information for herbicides evaluated in this study.

Herbicide	Mode of Action	Company	Doses evaluated (g a.i ha^−1^)	
			Sensitive population	Resistant population
Fenoxaprop-P-ethyl	ACCase Inhibitor	Jiangsu Agrochem Laboratory Co., Ltd.	8, 16, 32, 64, 128	32, 64, 128, 256, 512
Cyhalofop-butyl	ACCase Inhibitor	Jiangsu Fengshan Group Co., Ltd.	11.25, 22.5, 45, 90, 180	45, 90, 180, 360, 720
Penoxsulam7	ALS Inhibitor	Jiangsu Institute of Ecomones Co., Ltd.	2.8, 5.6, 11.25, 22.5, 45	2.8, 5.6, 11.25, 22.5, 45
Bispyribac-sodium	ALS Inhibitor	Jiangsu Institute of Ecomones Co., Ltd.	7.5, 15, 30, 60, 120	7.5, 15, 30, 60, 120
Pyraclonil	PPO Inhibitor	Hubei Xianghe Machinery Manufacturing Co., Ltd.	7.5, 15, 30, 60, 120	7.5, 15, 30, 60, 120
Quinclorac	Synthetic Auxin	Jiangsu Institute of Ecomones Co., Ltd.	47, 94, 188, 376, 752	47, 94, 188, 376, 752

ACCase, acetyl-CoA carboxylase; ALS, acetolactate synthase; PPO, protoporphyrinogen oxidase.

## 3 Results

### 3.1 Dose response to metamifop exposure

**Figure 1:**
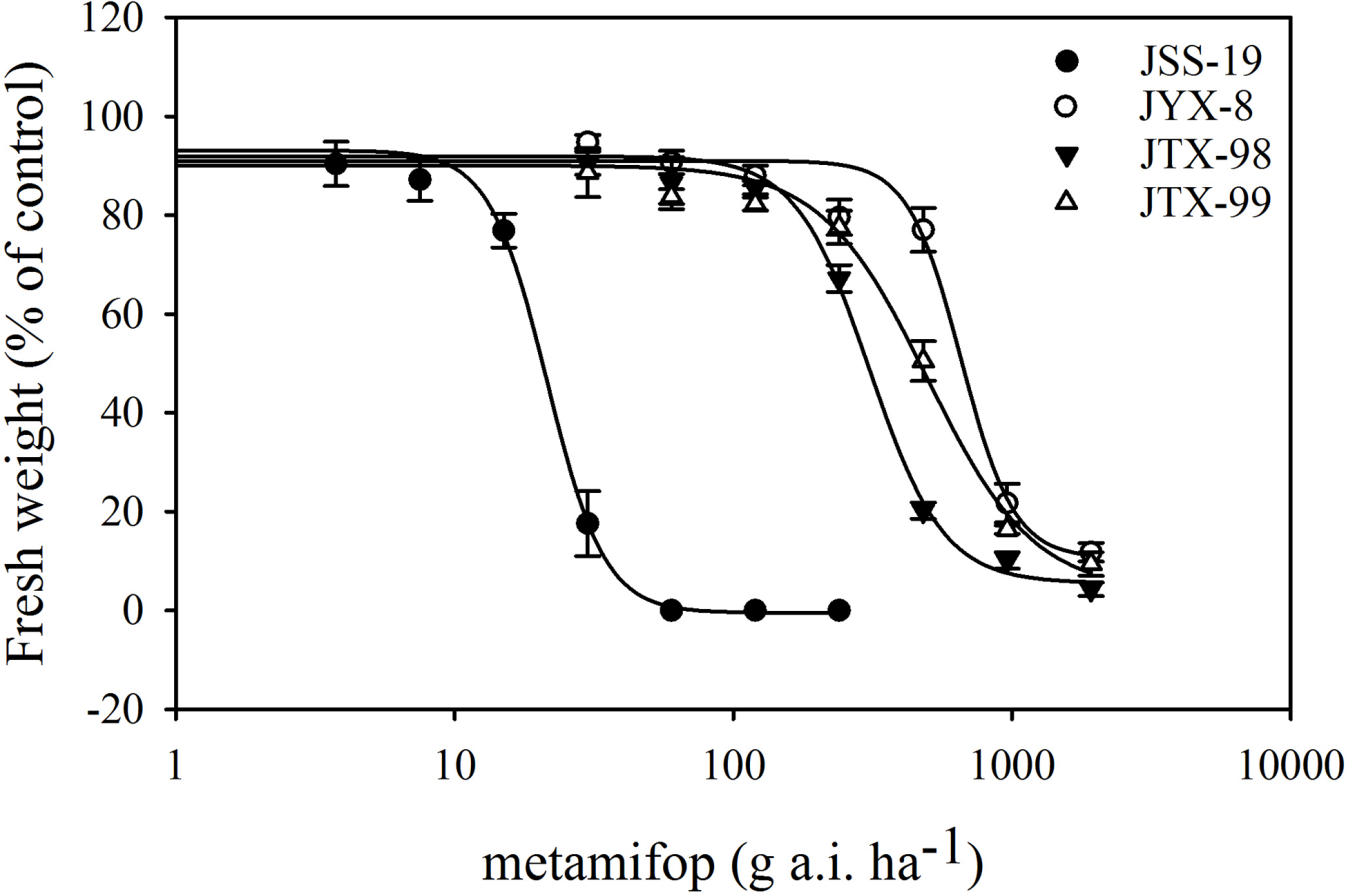
Dose response curves for four populations of *D*. *ciliaris* var. *chrysoblephara* to metamifop generated from dose response experiments. A log-logistic model was used to analyze the test data (the fresh weight percentage of the control). The GR_50_ values of resistant populations (JYX-8, JTX-98, and JTX-99) were significantly higher than in the sensitive population JSS-19. Vertical bars indicate standard errors.

Dose response experiments revealed that the JYX-8, JTX-98, and JTX-99 populations were resistant to metamifop (**Figure 1**; **Table 4**). Specifically, the GR_50_ values of these populations were much higher than the recommended dosage of metamifop (120 g a.i.ha^−1^). Moreover, their RI values were 30.64, 14.38, and 23.19, respectively.

**Table 4:** GR_50_ and RI values for four populations of *D*. *ciliaris* var. *chrysoblephara* exposed to metamifop in this study.

Population	GR_50_+SE (g a.i ha^−1^)	Susceptibility	RI
JSS-19	21.45 ± 0.79	S	–
JYX-8	657.22 ± 43.86	R	30.64
JTX-98	308.36 ± 14.14	R	14.38
JTX-99	497.38 ± 43.54	R	23.19

GR_50_, herbicide dose causing 50% growth reduction; SE, standard error; RI, GR_50_ value of resistant population divided by that of susceptible population; S, sensitive population; R, resistant population.

### 3.2 ACCase gene sequencing

The ACCase CT domain gene fragment amplified by the ACcp1/ACcp1R primers was 551 bp in length and contained a previously reported variable Ile/Leu codon at site 1,781. The gene fragment amplified by the primers ACcp4/ACcp2R was 406 bp in length and contained four previously known variable codons (Trp/Cys, Ile/Asn, Asp/Gly, and Gly/Ala) located at sites 2,027, 2,041, 2,078, and 2,096, respectively. After sequence alignment of gene fragments from the resistant and sensitive populations (**Table 5**), a single nucleotide substitution was observed from TGG to TGC that resulted in an amino acid substitution at position 2,027 from tryptophan in the susceptible population to cysteine in the resistant population JYX-8. No corresponding substitutions were observed for JTX-98 and JTX-99 ACCase gene.

**Table 5:** The mutation sites of ACCase gene in different *D*. *ciliaris* var. *chrysoblephara* populations.

Population	Ile1781	Trp2027	Ile2041	Gly2078	Gly2096
JSS-19	ATA	TGG	ATT	GAT	GGC
JYX-8	ATA	TGC	ATT	GAT	GGC
JTX-98	ATA	TGG	ATT	GAT	GGC
JTX-99	ATA	TGG	ATT	GAT	GGC

**Figure 2:**
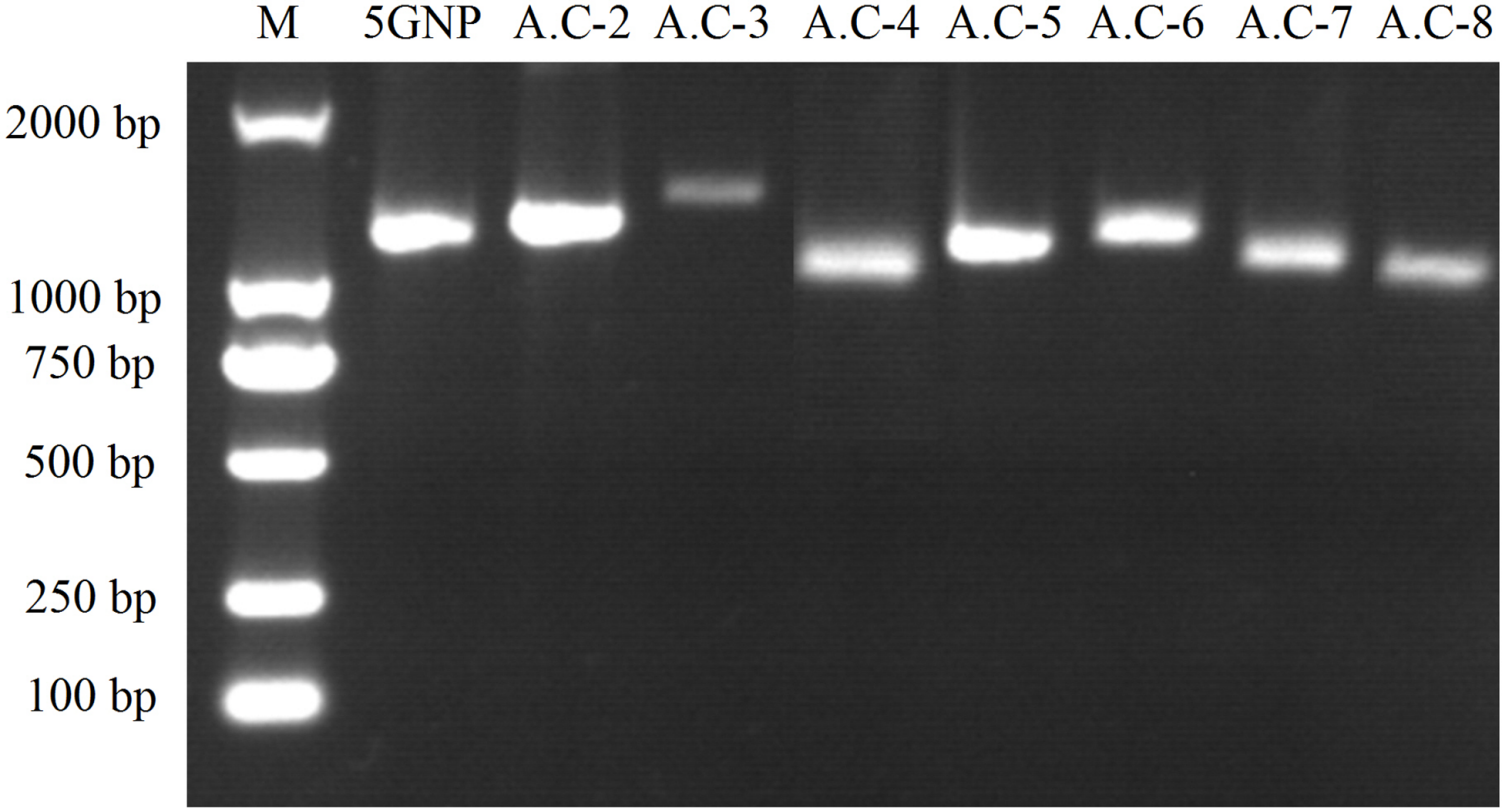
PCR amplification results for ACCase cDNA sequences. Eight primer pairs were designed based on homologous sequence comparison to amplify ACCase cDNA sequences *via* PCR and RACE. The sizes of the target fragments were 1,265, 1,304, 1,448, 1,136, 1,144, 1,232, 1,130, and 1,124 bp. The abbreviations above the image represent corresponding primers and the numbers on the left-hand side of the image indicate the size of DNA markers.

Using the extracted total RNA as template, eight primer pairs were designed using homologous sequence comparison to amplify ACCase cDNA sequences *via* PCR and RACE. The sizes of the target fragments amplified by the eight primer pairs were 1,265 bp, 1,304 bp, 1,448 bp, 1,136 bp, 1,144 bp, 1,232 bp, 1,130 bp, and 1,124 bp (**Figure 2**). The complete ACCase cDNA fragment was 7,277 bp in length and the ORF length was 6,966 bp. BLASTx analysis of the complete sequence against the NCBI database (**Table 6**) indicated the presence of amino acid sequence homology of 95.17%, 95.04%, 95%, and 94.10% between the ACCase encoded by the gramineae weeds *Panicum hallii*, *Setaria viridis*, *Setaria italica*, and *Echinochloa crus-galli*, respectively, suggesting that the amplified sequence was indeed an ACCase gene.

**Table 6:** BLAST results for *D*. *ciliaris* var. *chrysoblephara* ACCase proteins against the NCBI database.

Accession ID	Accession species	Maximum amino acid similarity
XP_025823701	*Panicum hallii*	95.17%
XP_034603265	*Setaria viridis*	95.05%
AAO62903.1	*Setaria italica*	95.00%
ADR32358.1	*Echinochloa crus-galli*	94.10%

### 3.3 Expression of ACCase gene

**Figure 3:**
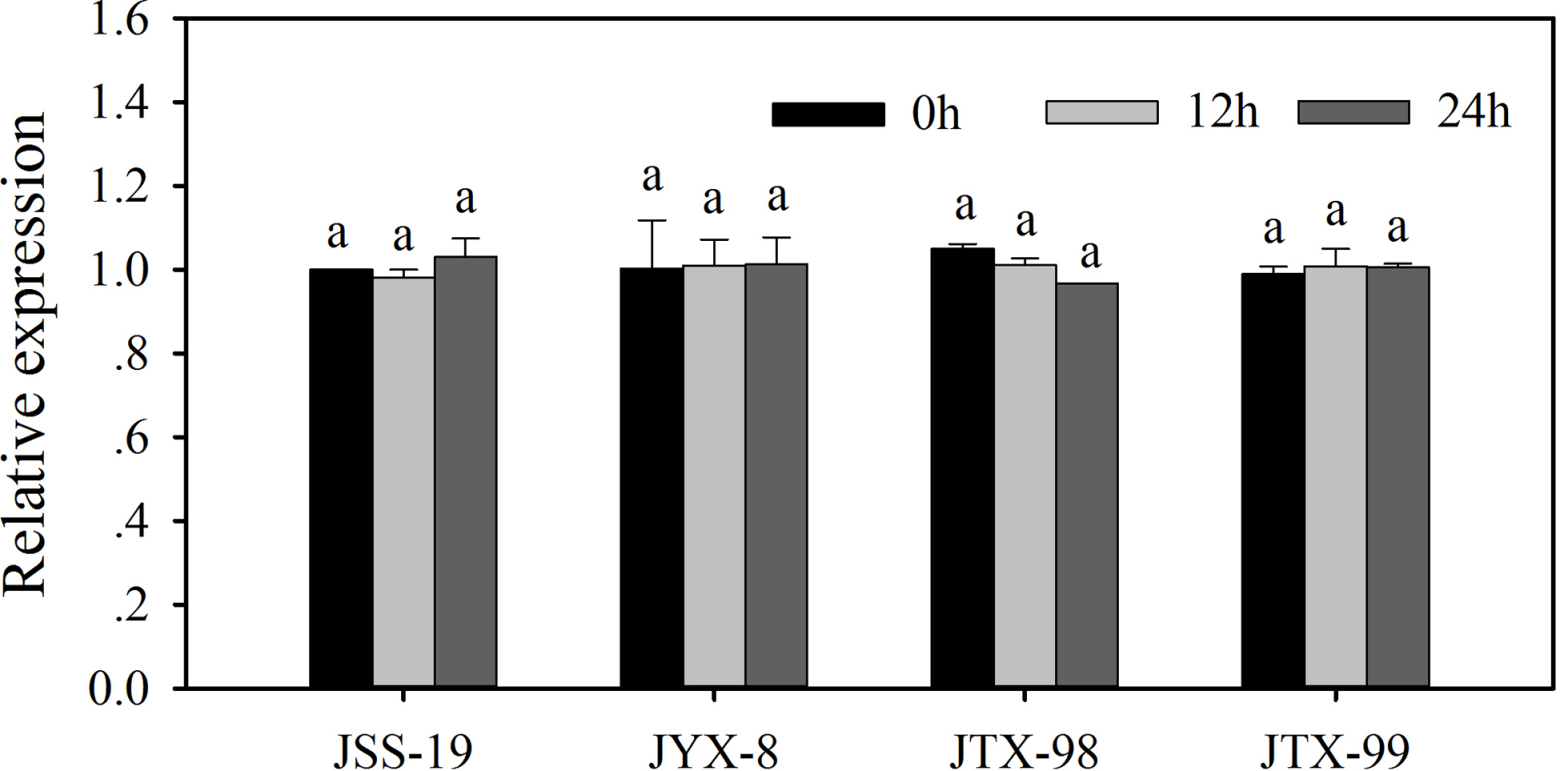
ACCase gene expression in four populations of *D*. *ciliaris* var. *chrysoblephara* after metamifop treatment. Gene expression levels were quantified by RT-qPCR and were not significantly different between control and metamifop treatments, both in the sensitive and resistant populations. Vertical bars indicate standard errors. The same lowercase letters indicate the lack of statistically significant differences based on Tukey’s tests.

The expression levels of ACCase gene in resistant and sensitive populations were quantified by RT-qPCR. Relative ACCase gene expression levels for each population are indicated as fold-level values compared to control assays in the sensitive JSS-19 population in the absence of metamifop (**Figure 3**). Expression levels were not significantly different between control and metamifop treatments, both in the sensitive and resistant populations. Thus, the resistance of the JYX-8, JTX-98, and JTX-99 populations to metamifop was not related to ACCase gene expression.

### 3.4 ACCase activity

**Figure 4:**
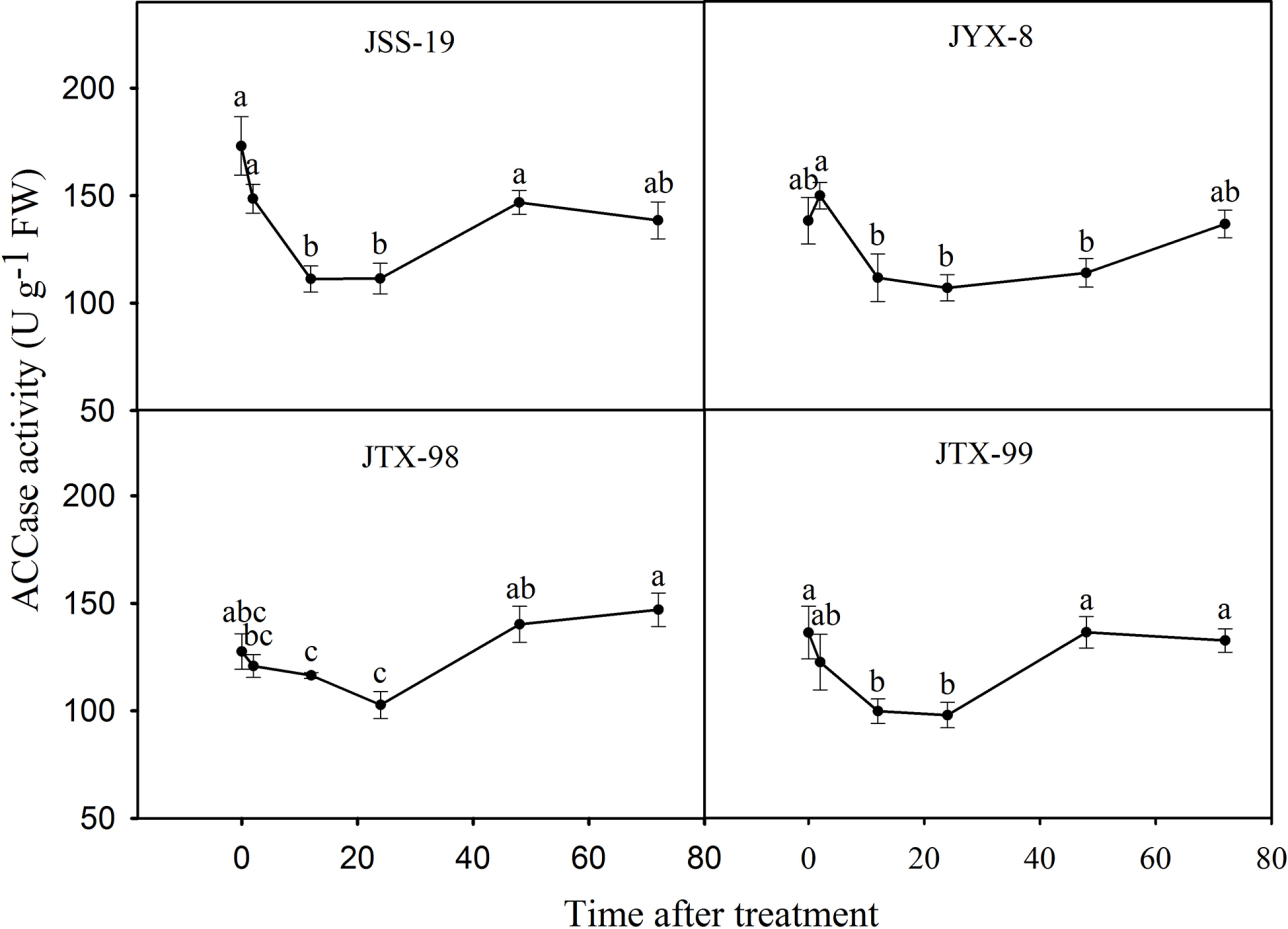
ACCase activities in four populations of *D*. *ciliaris* var. *chrysoblephara* after metamifop treatment. ACCase activities were determined using the ammonium molybdate spectrophotometric method. ACCase activities in resistant populations were less inhibited than in sensitive populations and also recovered to the same or even higher levels compared to untreated plants. Vertical bars indicate standard errors. Different lowercase letters indicate statistically significant differences (P < 0.05) based on Tukey’s tests.

The influence of metamifop to ACCase activity within four *D*. *ciliaris* var. *chrysoblephara* populations was determined using the ammonium molybdate spectrophotometric method. ACCase activities were not clearly different between 0 and 2 h, and then significantly decreased at 12 and 24 h, followed by a final gradual recovery at 48 and 72 h (**Figure 4**). The ACCase activity trends were similar between sensitive and resistant populations, although statistically significant differences remained. After treatment for 2 h, their activities were inhibited to 85.85%, 108.43%, 94.69%, and 89.93% of the control levels for the JSS-19, JYX-8, JTX-98, and JTX-99 populations, respectively. The lowest activities were observed at 24 h, with activities inhibited to 64.39%, 77.35%, 80.54%, and 71.84% levels of the controls, respectively. ACCase activity in the sensitive population JSS-19 was most inhibited across the above period. After treatment for 72 h, their activities were recovered to 80.04%, 98.87%, 115.24%, and 97.32% of control levels, respectively. ACCase activities returned to the same or even higher levels than in the control for the resistant populations, but only recovered to 80.04% of that of the control in the sensitive population. These results suggest that ACCase activities in resistant populations were less inhibited than in sensitive populations and recovered to the same or even higher levels compared to untreated plants.

### 3.5 Cross- and multi-resistance to other herbicides

Three metamifop-resistant populations of *D*. *ciliaris* var. *chrysoblephara* were cross-resistant to other ACCase-inhibiting herbicides (**Table 7**). Specifically, the JYX-8, JTX-98, and JTX-99 populations exhibited high resistance to cyhalofop-butyl, with GR_50_ values of 233.46, 157.8, and 185.06 g a.i.ha^−1^ and RI values of 21.83, 14.76, and 17.31, respectively. The populations also exhibited resistance to fenoxaprop-P-ethyl, with GR_50_ values of 174.47, 139.57, and 151.46 g a.i.ha^−1^ and RI values of 14.36, 11.79, and 12.47, respectively. Thus, the three resistant populations exhibited similar cross-resistance patterns to ACCase inhibitors.

**Table 7:** Sensitivity of *D*. *ciliaris* var. *chrysoblephara* to other herbicides.

Herbicide	Recommended dose (g a.i.ha^−1^)	Population	GR_50_+SE (g a.i.ha^−1^)	Susceptibility	RI
Cyhalofop-butyl	62.1	JSS-19	10.69 ± 0.15	S	–
		JYX-8	233.46 ± 5.51	R	21.83
		JTX-98	157.8 ± 18.27	R	14.76
		JTX-99	185.0 6 ± 7.5	R	17.31
Fenoxaprop-P-ethyl	105	JSS-19	12.15 ± 1.27	S	–
		JYX-8	174.47 ± 6.87	R	14.36
		JTX-98	139.57 ± 6.33	R	11.79
		JTX-99	151.46 ± 8.59	R	12.47
Penoxsulam	30	JSS-19	101.64 ± 4.20	R	/
		JYX-8	185.43 ± 14.95	R	/
		JTX-98	118.71 ± 4.56	R	/
		JTX-99	156.55 ± 11.73	R	/
Bispyribac-sodium	45	JSS-19	12.90 ± 1.82	S	–
		JYX-8	12.87 ± 1.74	S	1
		JTX-98	12.07 ± 0.77	S	0.79
		JTX-99	12.33 ± 0.87	S	0.85
Pyraclonil	210	JSS-19	69.90 ± 7.22	S	–
		JYX-8	112.10 ± 18.37	S	1.6
		JTX-98	89.22 ± 4.89	S	1.28
		JTX-99	80.61 ± 7.39	S	1.15
Quinclorac	375	JSS-19	93.67 ± 0.44	S	–
		JYX-8	86.83 ± 0.65	S	0.93
		JTX-98	93.70 ± 0.31	S	1
		JTX-99	96.65 ± 0.11	S	1.03

GR_50_, herbicide dose causing 50% growth reduction; SE, standard error; RI, GR_50_ value of resistant population divided by that of susceptible population; S, sensitive population; R, resistant population.

The multi-resistance characteristics of metamifop-resistant *D*. *ciliaris* var. *chrysoblephara* plants were also evaluated by whole-plant bioassays. The GR_50_ values of the four populations to the ALS inhibitor penoxsulam (101.64-185.43 g a.i.ha^−1^) were significantly higher than the recommended field dosage (30 g a.i.ha^−1^), suggesting the existence of multi-resistance to penoxsulam. The GR_50_ values of the resistant populations to the ALS-inhibiting herbicide bispyribac-sodium, the auxin mimic herbicide quinclorac, and the PPO-inhibiting herbicide pyraclonil were significantly lower than their recommended field dosages. In addition, the RI values were all< 2, suggesting the absence of multi-resistance to these herbicides.

## 4 Discussion

Jiangsu is one of the most important rice production areas of China. Metamifop has been used to control gramineae weeds in Jiangsu paddy fields for over 10 years. Consequently, metamifop use carries a high risk for herbicide resistance development. Consistently, we observed that continuous high-intensity use of metamifop did not mitigate the rapid spread of *D*. *ciliaris* var. *chrysoblephara* in paddy fields. We hypothesized that this lack of effect may be due to resistance of *D*. *ciliaris* var. *chrysoblephara* to metamifop. A high level of resistance to ACCase-inhibiting herbicides has been previously reported in various weed species including in wild oat (*Avena fatua* L.), Japanese foxtail (*Alopecurus japonicus*), and Amazon sprangletop [*Leptochloa panicoides* (J. Presl) Hitchc.] (Seefeldt et al., 1996; Xu et al., 2013; Tehranchian et al., 2016). *D*. *ciliaris* has only been previously observed to be resistant to ACCase herbicides *via* collection of plants from sod production fields in Georgia (Yu et al., 2017; Basak et al., 2019). In this study, *D*. *ciliaris* var. *chrysoblephara* populations collected from dry direct-seeding rice fields in the Jiangsu province were shown to exhibit high resistance to metamifop. ACCase inhibitors remain the primary herbicides used to control *D*. *ciliaris* var. *chrysoblephara*. However, the continued use of the same herbicides will inevitably lead to increased selection pressure, leading to rapid development of resistant populations and inestimable harm to rice production efforts.

The change of target enzyme activity caused by mutation or overexpression of target gene is the direct cause of weed resistance to herbicide. Herbicide efficacy is determined by the affinity of herbicides to enzymes. Physicochemical interactions are main factors that determine the affinity. For ACCase-inhibiting herbicides, their affinity and efficacy are determined by the interactions between the herbicides and the amino acids at specific positions of the polypeptide chain on ACCase CT domain. Thus, a single nucleotide mutation in the ACCase gene can result in amino acid substitutions imparting resistance to herbicides (Takano et al., 2020). Several amino acid substitutions of ACCase CT domain of grassy weeds have been reported to be involved in resistance to ACCase-inhibiting herbicides, including the Trp-2027-Cys substitution (Deng et al., 2020). Following the first report of the Trp-2027-Cys substitution in *Alopecurus myosuroides*, it has been observed in many weeds resistant to ACCase-inhibiting herbicides like *Avena sterilis*, *Lolium rigidum*, *Beckmannia syzigachne*, and *A*. *japonicus* (Delye et al., 2005; Liu et al., 2007; Yu et al., 2007; Xu et al., 2013; Pan et al., 2015). In this study, the Trp-2027-Cys substitution, resulting from a TGG to TGC nucleotide change, was observed in JYX-8 ACCase. Thus, the interaction between herbicide and target enzyme was influenced by target site mutation of ACCase gene and lead to the resistance of JYX-8 to metamifop. However, similar mutations were not observed for JTX-98 and JTX-99 populations. Overexpression of ACCase gene can confer resistance in large crabgrass to ACCase-inhibiting herbicides (Laforest et al., 2017). In this study, ACCase gene expression levels were not significantly different between control and metamifop treatments, both in the sensitive and resistant populations. ACCase activities were less inhibited in the resistant populations than in the sensitive population. Thus, ACCase from the resistant populations was much less sensitive to metamifop than the sensitive population (Xu et al., 2013). ACCase gene sequencing, gene expression, and enzyme activity investigations suggested that target-site resistance was the most likely mechanism conferring resistance in JYX-8, although the existence of NTSR mechanisms cannot be ruled out. A NTSR mechanism to metamifop, such as enhanced metabolism, may be present in JTX-98 and JTX-99, rather than a directed TSR. The unknown resistance mechanisms require further exploration.

The Trp-2027-Cys substitution can lead to cross-resistance to APP herbicides (Li et al., 2014), consistent with the results of this study. The JYX-8 population carrying the Trp-2027-Cys substitution was resistant to the APP herbicides cyhalofop-butyl and fenoxaprop-P-ethyl, with RI values of 21.83 and 14.36, respectively. In addition, no target site mutations were observed for the JTX-98 and JTX-99 populations, but they also exhibited cross-resistance to the APP herbicides cyhalofop-butyl and fenoxaprop-P-ethyl (RI values > 11.79) *via* unknown resistance mechanisms. The multi-resistance of the JYX-8, JTX-98, and JTX-99 populations to the ALS inhibitor penoxsulam was observed, but to the ALS inhibitor bispyribac-sodium, the auxin mimic quinclorac, and the PPO inhibitor pyraclonil was not observed. Bispyribac-sodium, quinclorac, and pyraclonil exerted good control on these populations. Thus, these herbicides with different modes of action could still be used to control metamifop-resistant *D*. *ciliaris* var. *chrysoblephara*.

Nevertheless, many studies have shown that different resistance mechanisms (i.e., TSR and/or NTSR mechanisms) may be present in a single weed population that is resistant to a variety of herbicides (Beckie et al., 2012). Multiple target-site resistance to ACCase and ALS inhibitors has been observed in black-grass (Bailly et al., 2012). Further, glyphosate-resistant rigid ryegrass exhibited multiple resistance to ACCase- and ALS- inhibiting herbicides. Specifically, ALS resistance in the population was due to an insensitive target enzyme, while ACCase resistance was due to a non-target-site mechanism (Neve et al., 2004). Consequently, control methods should be carefully evaluated for weed populations with different resistance mechanisms. No evidence of non-target-site resistance has been shown for *Digitaria* spp. weeds. However, the resistant populations JTX-98 and JTX-99 investigated in this study likely exhibit NTSR mechanisms, warranting additional attention to control methods.

In conclusion, three populations of *D*. *ciliaris* var. *chrysoblephara* collected from dry direct-sown rice fields of the Jiangsu province have evolved resistance to metamifop, and the target-site mutation Trp-2027-Cys is responsible for resistance in the JYX-8 population. To our knowledge, this study represents the first evidence for a molecular mechanism of *D*. *ciliaris* var. *chrysoblephara* resistance to metamifop. In addition, cross- and multi-resistance patterns to other herbicides were also investigated in this study. Non-target-site resistance mechanisms cannot be presently ruled out for the resistance of *D*. *ciliaris* var. *chrysoblephara* to metamifop. Consequently, additional studies are needed to comprehensively understand the mechanism underlying the resistance traits evaluated here.

## Data availability statement

The original contributions presented in the study are included in the article/supplementary material. Further inquiries can be directed to the corresponding author.

## Author contributions

HW and YL conceived and designed the experiments; JC and YT performed the lab work, data acquisition, and data analysis; JC and YT wrote the manuscript; TG assisted in performing the experiment; ZZ and GL contributed to the discussion and reviewed the manuscript. All authors contributed to the article and approved the submitted version.
